# Osteosarcoma in the Setting of Genetically Confirmed *IFITM5-*Related Osteogenesis Imperfecta

**DOI:** 10.1155/crip/3377588

**Published:** 2026-05-30

**Authors:** Ava McKane, Morgan Noel, Andrew Hiatt, Cynthia Emory

**Affiliations:** ^1^ Department of Orthopaedic Surgery and Rehabilitation, Wake Forest University School of Medicine, Winston-Salem, North Carolina, USA, wakehealth.edu; ^2^ Department of Pathology, Wake Forest University School of Medicine, Winston-Salem, North Carolina, USA, wakehealth.edu

**Keywords:** case report, *IFITM5*, osteogenesis imperfecta, osteosarcoma

## Abstract

**Background:**

Osteogenesis imperfecta (OI) is a group of skeletal dysplasias characterized by recurrent fractures and fragile bones. Two pathogenic variants in the *IFITM5* gene cause distinct forms of OI: The recurrent c.–14C>T variant causes OI type V (MIM #610967), characterized by hyperplastic callus formation and interosseous membrane ossification, while the rare c.119C>T (p.Ser40Leu) variant causes a phenotypically distinct, severe form of *IFITM5*‐related OI without these hallmark features. Radiographically, OI‐related skeletal lesions may resemble malignant bone tumors such as osteosarcoma, leading to diagnostic challenges. Although the co‐occurrence of OI and osteosarcoma has been reported, with approximately nine documented cases, all involved other OI subtypes. No case of true osteosarcoma arising in a patient with genetically confirmed *IFITM5*‐related OI has been previously described. We present this case to highlight the novelty of this association, improve recognition of the distinguishing features, and prevent future misdiagnosis.

**Case Presentation:**

A 23‐year‐old female with *IFITM5*‐related OI, confirmed by molecular testing showing heterozygosity for a pathogenic *IFITM5* mutation (c.119C>T, p.Ser40Leu; parental testing to determine inheritance was not performed) presented to our Comprehensive Cancer Center in May 2025 with localized groin pain, later diagnosed as a nondisplaced pelvic fracture. Although initial clinical and radiographic findings were consistent with the patient′s underlying OI, further evaluation revealed features concerning for concomitant osteosarcoma. The patient was treated with the MISER chemotherapy protocol with subsequent surgical resection.

**Conclusion:**

Distinguishing OI‐related skeletal changes from malignant bone tumors requires integration of clinical history, genetic testing, and careful pathologic evaluation via a multidisciplinary approach. While prior reports of concomitant osteosarcoma and OI have involved other subtypes, this case represents the first documented true co‐occurrence in a patient with *IFITM5*‐related OI and underscores the importance of recognizing the overlapping and divergent features of these conditions when rendering a diagnosis and initiating treatment protocols.

## 1. Introduction

Osteogenesis imperfecta (OI) is one of the most common forms of heritable skeletal dysplasia. The Osteogenesis Imperfecta Foundation estimates that between 20,000 and 50,000 individuals in the United States are affected by OI [1]. There are more than 20 recognized forms of OI, colloquially known as “brittle bone disease,” characterized by increased risk of fracture, bone deformity, and growth deficiency [2, 3]. While more than 80% of cases of OI result from dominant pathogenic variants in COL1A1 or COL1A2, a subset involves noncollagen genes.

Unlike classical types of OI caused by type I collagen defects, other forms are associated with variants in the interferon‐induced transmembrane protein 5 (*IFITM5)* gene [4]. Two distinct pathogenic variants in *IFITM5*, which encodes bone‐restricted interferon‐induced transmembrane protein‐like (BRIL), cause phenotypically distinct forms of OI. The recurrent c.–14C>T variant in the 5 ^′^‐UTR causes OI type V (MIM #610967), a moderate to severe form of OI. OI type V is distinguished by hyperplastic callus formation after fractures, calcification of interosseous membranes, and radial head dislocation [5]. This variant acts through a gain‐of‐function mechanism, disrupting osteochondroprogenitor differentiation and mineralization pathways, contributing to abnormal cartilage and bone development [6]. In contrast, the c.119C>T (p.Ser40Leu) variant causes a different phenotype—a severe form of OI characterized by extreme short stature and severe skeletal deformity, but without the hallmark radiographic features of OI type V. This variant is exceedingly rare, as only 10 patients with this variant have been reported to date [7].

Osteosarcoma is the most common primary malignant bone tumor. It arises from mesenchymal stem cells and produces an osteoid matrix. It has a bimodal age distribution, with peak incidence in adolescents and again in older adults, and typically involves the metaphyses of long bones, especially the distal femur and proximal tibia. Osteosarcoma presents clinically as progressive pain that worsens with activity, a tissue mass tender to palpation, decreased range of motion, and susceptibility to pathologic fracture [8].

The co‐occurrence of osteosarcoma in patients with OI is rare, with only nine cases reported. All documented cases with confirmed osteosarcoma have involved Types I, III, and IV [9, 10]. There have been reports of suspected osteosarcoma in patients later diagnosed with OI, where lesions mimicking malignancy were ultimately identified as benign hyperplastic callus formation [11]. This highlights the diagnostic challenge in distinguishing between these entities. To our knowledge, this case report presents the first documented case of osteosarcoma in a patient with *IFITM5*‐related OI, though there have been reports of OI type V mimicking osteosarcoma [11]. We aim to highlight the diagnostic, pathophysiologic, and management considerations in this rare clinical scenario to help clinicians differentiate between similar‐appearing conditions and avoid misdiagnosis.

## 2. Case Presentation

**Figure 1:**
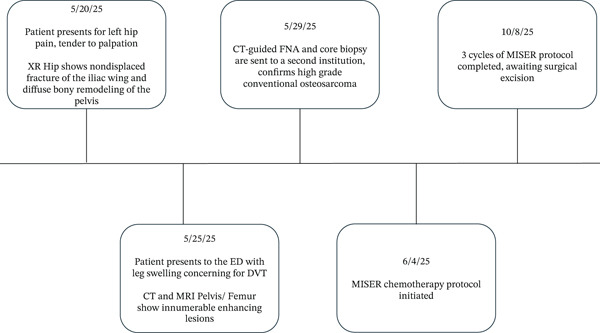
Timeline from symptomatic presentation to current treatment status, including diagnosis and initiation of corresponding treatment, over the course of 141 days.

**Figure 2:**
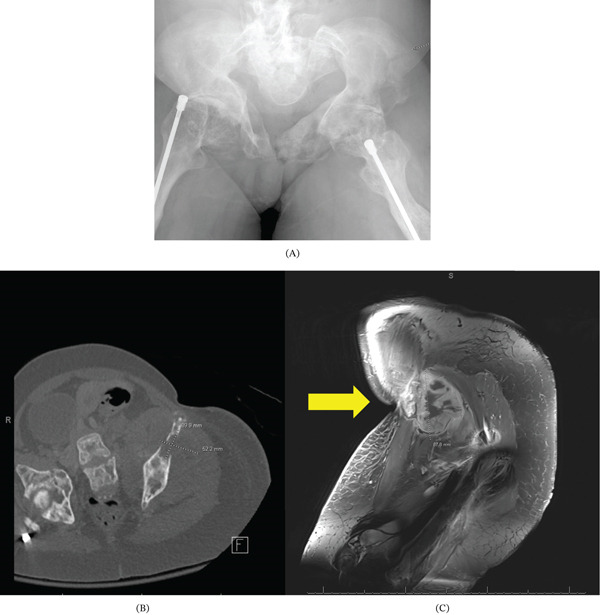
(A) XR pelvis demonstrating a nondisplaced fracture of the iliac wing with bony remodeling. (B, C) CT lower extremity and MRI pelvis demonstrating a heterogeneously enhancing extraosseous mass arising from the ilium.

A 23‐year‐old wheelchair‐dependent female with *IFITM5*‐related OI, confirmed by molecular testing showing heterozygosity for pathogenic *IFITM5* mutation (c.119C>T, p.Ser40Leu; parental testing to determine inheritance was not performed) presented to the orthopedic clinic with localized groin pain that was tender to palpation and progressively worsened throughout the day (Figure 1). She reported no specific incident of trauma or injury. Initial radiographs revealed an acute, nondisplaced fracture of the iliac wing with bony remodeling consistent with underlying OI (Figure 2A). The patient′s medical history was significant for preterm birth and vitamin D deficiency. Family history is significant for carcinoid tumor, colon cancer, lung cancer, multiple myeloma, and prostate cancer in five separate relatives.

Five days later, the patient presented to the Emergency Department (ED) with a tight sensation and swelling in the leg, raising concern for deep vein thrombosis (DVT). Although Doppler ultrasound findings were negative for DVT, subsequent CT imaging of the lower extremity (Figure 2B) and MRI of the pelvis/femur (Figure 2C) revealed a heterogeneously enhancing soft tissue lesion concerning for malignancy such as Ewing sarcoma or osteosarcoma, and innumerable enhancing lesions throughout the skeleton, which could have represented marrow changes from OI or potentially metastatic disease.

**Figure 3:**
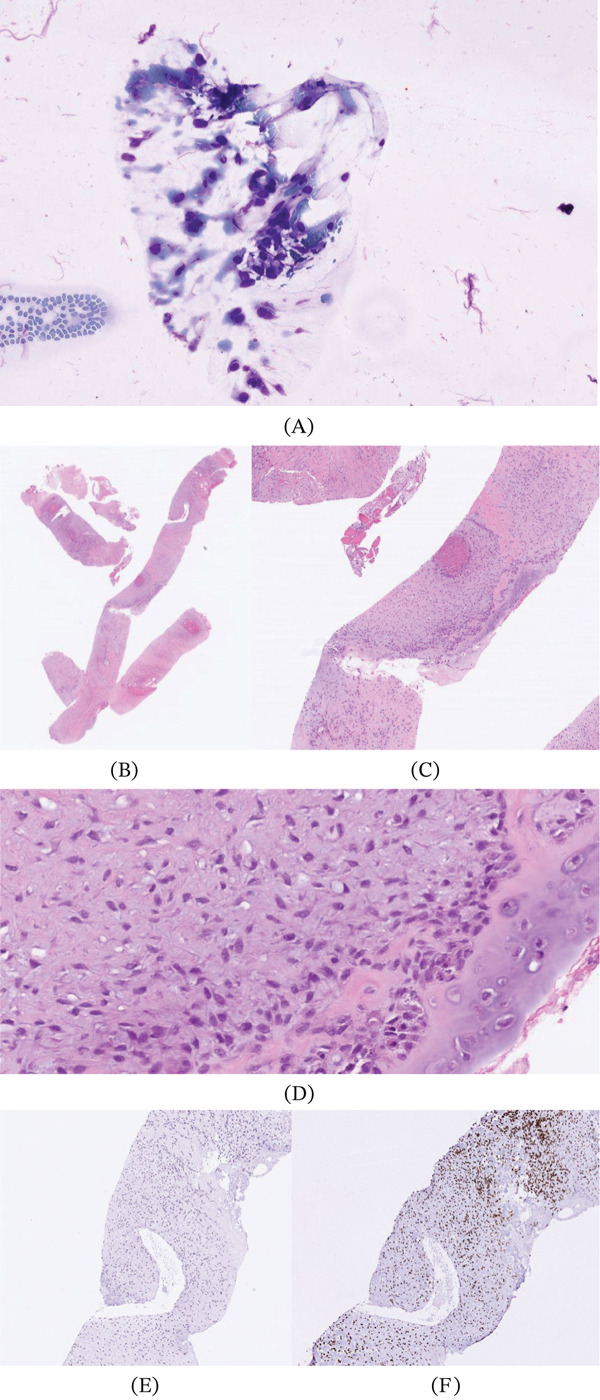
(A) Touch prep of the left iliac crest demonstrates atypical cells with hyperchromatic nuclei, increased nuclear‐to‐cytoplasmic (N:C) ratio, and are loosely cohesive. (B–D) Core biopsy of the left iliac crest with H&E whole slide digital images increasing in power and labeled from left to right. (B) Fragments of bone filled with hypercellular areas of blue cells and focal areas of pink necrosis. (C) Higher power emphasizing an area of necrosis with surrounding neoplastic cells replacing the osteoid matrix. (D) Even higher power highlighting normal cartilage on the right of the image with the plasmocytic, ovoid, malignant cells with hyperchromatic nuclei filling the rest of the photomicrograph. (E, F) Immunohistochemical stains. (E) The malignant cells are cytokeratin AE1/AE3 negative, which is an epithelial marker. (F) The malignant cells are SATB2 positive, which is an osteoblast lineage marker. These stains highlight that the malignant cells are of osteoblastic lineage supporting the diagnosis of osteosarcoma.

Given the radiographic suspicion for neoplasm, orthopedic oncology was consulted. Image‐guided biopsy of the pelvic lesion was performed, revealing a tumor most consistent with osteosarcoma, demonstrating neoplastic bone and cartilage erosion with an associated osteoid matrix (Figure 3A, B, C, D, E and F). A second opinion from an outside institution confirmed the diagnosis of high‐grade conventional osteosarcoma.

As a result of these findings, the patient began multiagent chemotherapy with the MISER protocol consisting of ifosfamide, doxorubicin, cisplatin, and methotrexate, an established protocol for high‐grade osteosarcoma. At follow‐up with orthopedics approximately 1 month following the initiation of chemotherapy, the patient reported marked improvement in pain. At 2 months postdiagnosis, pain improvement persisted, but evaluation revealed incomplete bladder emptying with mild hydronephrosis, as well as partially occlusive acute DVT. The bladder symptoms were addressed with tamsulosin, a sympatholytic bladder muscle relaxant, and the DVT was managed with apixaban, a direct oral anticoagulant, both of which led to resolution of the patient′s symptoms. At 4 months posttreatment initiation, the patient received the third cycle of chemotherapy, which was well‐tolerated except for pedal edema. This was managed with furosemide, which resulted in the resolution of symptoms. The patient underwent surgical resection of her tumor following neoadjuvant chemotherapy.

## 3. Discussion

One major diagnostic challenge in OI is distinguishing new pathologic lesions from underlying skeletal disease. In patients with the classical OI type V (recurrent *IFITM5* c.–14C>T variant [MIM #610967]), the frequent development of hyperplastic callus can present as a rapidly enlarging, expansile bone mass that closely mimics both the radiographic and clinical presentations of osteosarcoma [12]. Both conditions may demonstrate aggressive periosteal reactions, such as a sunburst appearance, and soft tissue swelling on imaging. Histologically, the presence of irregular trabeculae of woven bone and fibrovascular stroma can resemble the malignant osteoid appearance of osteosarcoma, especially in suboptimal biopsy samples [13]. Notably, the p.Ser40Leu variant identified in this patient yields a phenotypically distinct form of *IFITM5*‐related OI that lacks these hallmark features of OI type V. Nonetheless, given the overlapping features across OI subtypes, accurate diagnosis requires careful integration of imaging, histopathology, and clinical history. A multidisciplinary review is essential to ensure that pathological and radiographic findings are interpreted within the context of the patient′s clinical presentation, thereby minimizing the risk of misdiagnosis.

The occurrence of osteosarcoma in OI is incredibly rare. Most reported cases of suspected osteosarcoma in OI type V represent misdiagnosed hyperplastic callus rather than a true malignancy. All confirmed cases of co‐occurring osteosarcoma and OI have involved Types I, III, and IV. This case therefore represents a particularly unique and diagnostically challenging scenario—the first documented osteosarcoma in a patient with *IFITM5*‐related OI. As shown in previous literature, the absence of genetic testing or family history of OI may preclude clinicians from recognizing a diagnosis of OI [14]. In this patient, the repeated history of fractures since birth and genetic confirmation of a heterozygous pathogenic variant in the *IFITM5* gene supported the diagnosis of OI. However, this diagnosis complicated the interpretation of the lesion, necessitating the reliance on biopsy findings and prompting a second opinion before making a final diagnosis.

Management of osteosarcoma in the context of *IFITM5*‐related OI is complicated by inherent bone fragility. Bisphosphonate therapy is commonly used to reduce fracture risk and increase bone mineral density in OI and has demonstrated benefit in cases of OI type V. Zeitlin et. al found that pamidronate therapy in patients with OI type V led to an 86% increase in cortical thickness and a reduction of fracture incidence from 1.5 fractures per year to 0.5 [15]. However, bisphosphonate use must be carefully managed in the context of concurrent osteosarcoma. These agents can impair bone healing, particularly when combined with cytotoxic chemotherapy, emphasizing the importance of accurate diagnosis before initiating treatment. There is no clear evidence from randomized studies demonstrating a clinical benefit for the use of bisphosphonates in osteosarcoma outcomes, which underscores the need for individualized treatment planning [16]. Patients must be closely monitored to minimize complications, such as delayed union, malunion, or additional fractures.

In this case, bisphosphonate therapy was discontinued 1 week after the patient′s initial presentation of hip pain and later resumed receiving zoledronic acid as supportive therapy for the underlying OI approximately 1 month after the start of chemotherapy (MISER protocol). This case is limited by the absence of long‐term follow‐up data and the lack of parental testing to determine de novo status of the *IFITM5* variant. Extended monitoring is necessary to evaluate the effectiveness of the MISER protocol and bisphosphonate therapy in a patient with co‐occurring *IFITM5*‐related OI and osteosarcoma.

## Author Contributions

All authors contributed to the article and approved the submitted version.

## Funding

No funding was received for this manuscript.

## Consent

Informed consent for publication of the clinical details and accompanying images was obtained from the patient described in this case report.

## Conflicts of Interest

The authors declare no conflicts of interest.

## Data Availability

Data sharing is not applicable to this article as no datasets were generated or analyzed during the current study.
